# Niche conservatism in *Gynandropaa* frogs on the southeastern Qinghai-Tibetan Plateau

**DOI:** 10.1038/srep32624

**Published:** 2016-09-07

**Authors:** Junhua Hu, Olivier Broennimann, Antoine Guisan, Bin Wang, Yan Huang, Jianping Jiang

**Affiliations:** 1 CAS Key Laboratory of Mountain Ecological Restoration and Bioresource Utilization & Ecological Restoration Biodiversity Conservation Key Laboratory of Sichuan Province, Chengdu Institute of Biology, Chinese Academy of Sciences, Chengdu 610041, China; 2 Department of Ecology and Evolution, University of Lausanne, 1015 Lausanne, Switzerland; 3 Institute of Earth Surface Dynamics, University of Lausanne, 1015 Lausanne, Switzerland

## Abstract

The role of ecological niche in lineage diversification has been the subject of long-standing interest of ecologists and evolutionary biologists. *Gynandropaa* frogs diversified into three independent clades endemic to the southeastern Qinghai-Tibetan Plateau. Here, we address the question whether these clades kept the same niche after separation, and what it tells us about possible diversification processes. We applied predictions in geographical (G)-space and tests of niche conservatism in environmental (E)-space. Niche models in G-space indicate separate regions with high suitability for the different clades, with some potential areas of sympatry. While the pair of central and eastern clades displayed the largest niche overlap for most variables, and strict niche equivalency was rejected for all clade-pairs, we found no strong evidence for niche divergence, but rather the signature of niche conservatism compared to null models in E-space. These results suggest a common ancestral ecological niche, and as such give good support to divergence through allopatric speciation, but alternative explanations are also possible. Our findings illustrate how testing for niche conservatism in lineage diversification can provide insights into underlying speciation processes, and how this information may guide further research and conservation practices, as illustrated here for amphibians on the Qinghai-Tibetan Plateau.

Whether ecological factors promote lineage diversification and speciation has been an outstanding question in ecology and evolutionary biology during the last decade^1^,^2^,^3^,^4^,^5^. It was initially proposed that niches tend to be conserved in the sense that descendant species tend to inhabit similar geographical areas or ecological niches to their immediate ancestors^6^,^7^,^8^. By conservatism, we mean specifically here that niches tend to remain more similar to the one of their ancestor than to those of randomly chosen more distant species, not that they must remain strictly equivalent^9^. The evidence for niche conservatism appears at first glance to be mixed in the literature, but a closer look reveals that the evidence for niche conservatism highly depends on the niche test being used (i.e. niche similarity versus niche equivalency tests)^10^ and on the temporal frames of the study systems in question^8^,^10^. While niche equivalency may be useful to test for the transferability of niche models in space and time, niche similarity tests make more sense to test biogeographic and evolutionary hypotheses^8^. Recent and short-term events, such as species invasions or distributional shifts over relatively short time frames, show considerable tendencies towards conservatism. Longer-term events, on the other hand, such as differentiation across phylogenies, show a tendency for a breakdown in conservatism^8^,^11^. Overall, the relative importance of niche conservatism versus niche divergence for influencing lineage diversification in taxa crossing heterogeneous habitats and putative geographical barriers remains poorly understood^8^,^11^,^12^,^13^.

Quantifying niche differentiation among closely related and parapatric taxa is of fundamental interest for ecologists and evolutionary biologists, since this quantification provides a solid basis for further experimental and observational work and raises questions about the “mechanistic underpinnings of broad-scale geographic patterns”^14^ (see also^6^,^15^,^16^,^17^,^18^). The development of ecological niche models coupled with geographic information systems (GIS) and multivariate analyses in environmental space^10^ (hereafter ENMs sensu lato) renewed and strengthened the interest in ecological niches^3^,^8^,^10^,^19^,^20^. Being rooted deeply in niche theory, ENMs make it possible to quantify niches at unprecedented scales across space and time^15^. ENMs can be applied widely to clarify questions in ecology and evolution by assessing niche similarity between taxa and by projecting the niches of taxa in response to environmental changes^21^,^22^,^23^.

However, when assessing niche similarity from occurrence records that are spatially representative of the distribution of species, new conceptual and statistical challenges emerge. For example, underlying most ENMs, the environmental data (e.g. temperature) are often spatially correlated, potentially confounding meaningful niche divergence with geographic distance^20^. This problem of spatial autocorrelation is unavoidable but can be addressed by using null models when testing niche conservatism versus divergence^9^,^20^. To meet the increasing need for robust methods for understanding niche differences in evolutionary and community contexts, realized environmental niches can be directly quantified and compared in the environmental (E)-space using multivariate statistics^10^. A recent ordination null test applies a kernel estimation to smoothen inevitable sampling effort biases and can test hypotheses regarding niche conservatism^10^.

The Qinghai-Tibetan Plateau (QTP) and its adjacent areas is an interesting region for evaluating the use of niche tests to uncover lineage diversification processes. It spans three biodiversity hotspots: Himalaya, Indo-Burma and the mountains of southwestern China^24^. The uplifting of the QTP during the Late Cenozoic released novel ecological opportunities for explosive diversification and created substantial inter- and intraspecific genetic heterogeneity, forming a model ecosystem for investigating lineage diversification and speciation^25^,^26^,^27^,^28^,^29^. The southeastern QTP is characterized by a landscape of river gorges and steep mountain ridges with a series of parallel alpine ridges and rivers running north to south. Such topographical complexity within a rather small geographical region led to dramatic ecological stratification and environment heterogeneity^30^. Moreover, this region has a classic montane climate with striking vertical climatic zonation, ranging from the subtropical to the nival zones, with diverse vegetation types and landscapes^30^. However, this region is understudied compared to other regions, e.g. the European Alps, the Rocky Mountains and the Appalachian Range^31^,^32^, notwithstanding its diverse environmental conditions that harbor one of the most diversified fauna and flora in the world^26^,^33^ and its notoriously endangered ecosystems^24^. Thereby, the southeastern QTP is a well suited region for inquiring into the inertial tendency of organisms to maintain their current ecological niche (conservatism) and the effects of natural selection on populations, which differ in habitat across ecological landscapes.

Ectotherms such as amphibians, which are known to have evolved slowly and conservatively, are well suited for assessing the role of environment and geography on lineage diversification because of their low vagility and strong responses to environmental factors^19^,^31^,^34^. Dicroglossid frogs in the genus *Gynandropaa* originated and diversified on the southeastern QTP^25^,^26^,^27^, and occur in southwestern China (Sichuan, Yunnan and Guizhou provinces), northern Vietnam and Myanmar and presumably in intervening Laos (Fig. 1a,b)^26^,^35^. With a low dispersal capability, they mainly reside in rocky streams among mountain forests at low-medium elevations (about 600–2900 m)^26^,^29^. Adult males are characterized by two patches of keratinized spines on the chest during breeding seasons^26^. However, the taxonomy of these frogs has been subject to debate, even in the genus-level classification, with several generic and subgeneric shifts^25^,^26^,^27^. All these frogs had been classified under the genus *Paa* and/or *Rana* for decades^26^,^35^; thereafter, Che *et al*.^36^ suggested to categorize them into the genus *Nanorana* that has been adopted by Frost^35^; while recently, the genus *Gynandropaa* is more widely accepted^26^,^27^,^37^,^38^. Additionally, due to high similarity in morphology (e.g. body size), the taxonomic recognitions of species in this genus are controversial, involving six named species (i.e. *yunnanensis, phrynoides, liui, sichuanensis, bourreti* and *feae*)^26^,^27^,^35^ (see also Table S1 in Huang *et al*.^38^). Zhang *et al*.^25^ categorized all taxa in this genus into a single species, but they identified three major independent evolutionary clades called W (western), C (central), and E (eastern) based on genealogical analyses of mitochondrial DNA (see also Wang^27^). Integrating morphological characteristics, phylogenetic relationships (inferred by maximum likelihood and Bayesian inference methods) and geographical distribution patterns, Huang *et al*.^38^ proposed that the clades W, C and E correspond to *Gynandropaa yunnanensis, G. sichuanensis* and *G. phrynoides*, respectively^38^. To avoid potential debate in classification, we use the generic taxonomy of *Gynandropaa*^37^, and the three deep-divergent clades rather than the newly recognized species^25^,^27^,^38^. According to previous studies on the phylogeography of *Gynandropaa*^25^,^27^, long-term geographic isolation among clades being formed in the Early Pliocene and Early Pleistocene promoted allopatric speciation in this genus. The estimated divergence times of about 4.8–5.3 Ma are in concert with the uplifting of the QTP^25^,^27^. Different clades are concordant with different geographical regions: W in the Hengduan Mountains region; C in the Chuxiong Basin and the southwestern Sichuan Plateau; and E on the central and eastern Yunnan Plateau^25^,^38^. The historical range expansions of clades probably occurred early in the Middle Pleistocene accompanying dramatic climatic oscillations^25^,^27^. These would have resulted in secondary contact of previously allopatric populations. Co-occurrences between clades currently occur in locations where different rivers conjoined historically (co-occurring of C-E in Luquan, Lufeng and Wuding, and of W-C syntopic in Binchuan; Fig. 1b). However, restricted gene flow between clades was strongly implied and long-term genetic isolation among clades was corroborated^25^. Within the E-space encircled by annual mean temperature and annual precipitation, clade W lives in wetter areas than clades C and E (Fig. 1c). The three clades closely resemble each other ecologically and phenotypically and their diversification is argued to be driven by sexual selection rather than natural selection^26^,^27^. The parapatric ranges between clades and allopatric speciation in *Gynandropaa*^25^,^27^ provide opportunities to investigate the role of niche conservatism in the diversification process of this genus thoroughly^9^,^20^.

In this study, we address the question whether *Gynandropaa* clades kept the same niche after separation, and accordingly explore the potential role of niche conservatism in their diversification process. Specifically, based on large-scale environmental variables in concert with occurrence data, we first constructed ecological niche models (ENMs) for each clade using a maximum entropy algorithm^39^. Next, using the observed occurrences and environmental data, we explored the question of whether these closely related clades inhabit more similar environments than expected based on background (southeastern QTP) environmental divergence by a ordination null test of PCA-env in environmental (E)-space^23^. We finally used ENM predictions in geographical (G)-space to test for strict niche equivalency^9^,^40^, providing a strict niche comparison among clades^41^ (expected to be rejected for any pair of distinct species^10^). Combined with the phylogeographic relationships^25^,^27^, the results of these analyses provide a broad and multifaceted view of niche variation and differentiation in the *Gynandropaa* frog genus and provide further insights into the possible diversification processes within dicroglossid frogs.

## Results

### Niche modeling and projections

The ENMs had a great predictive accuracy as measured by the area under the receiver operating characteristic curve (AUC) metric (AUC_training_: 0.963–0.979; AUC_test_: 0.947–0.963). All three clades (W, C and E) were predicted to occur in mild and humid conditions, with high temperatures in *T*_war_ (mean temperature of the warmest quarter; 20.4–21.0 °C) and relatively high amount of precipitation in the wettest month (Prec_*wet*_; 196.9–288.1 mm). T_*anu*_ (annual mean temperature) ranged from 15.4° to 16.9 °C. Additionally, striking variations occurred in a number of environmental dimensions, with clades C and E being at the low end of the spectrum for a number of variables [T_*anu*_, T_*iso*_ (isothermality), T_*dry*_ (mean temperature of the driest quarter), T_*war*_, Prec_*wet*_, Prec_*dry*_(precipitation of the driest month), AET_*anu*_ (annual actual evapotranspiration) and alpha (Priestley-Taylor alpha coefficient, generalized as the ratio of AET_*anu*_ over annual potential evapotranspiration); Table S1].

Clearly delimited separate regions with high environmental suitability were predicted for different clades: mostly in the west of the Lancang-Mekong river for clade W, scattered areas surrounding the Red river and its tributaries for clade C, and the region between the Pudu river (a major tributary of the Jinsha river) and the Nanpen river (the headstream of the Pearl river) for clade E (Fig. 2a–c). Substantial potential sympatry in geographical space was predicted in the clade pairs C-E (*c.* 4.16*10^4^ km^2^) and W-C (*c.* 1.86*10^4^ km^2^), while sympatry was narrow in the pair W-E (*c.* 3671 km^2^) and across clades (*c.* 2195 km^2^; Fig. 2d).

### Niche comparisons based on observed occurrences in E-space

The ordination approach using PCA-env^10^ revealed the niche patterns for each clade pair in E-space under the background defined by areas predicted as present in the ENMs binary predictions (Figs 3 and S1). Niche overlap of the clade pair C-E was higher than that of both the pairs C-W and W-E. For the pair of C-E, ordination null tests of niche similarity showed that niches were more similar than random in two reciprocal directions (Figs 3a and S1a). However, for both pairs C-W and W-E, observed niche overlap values constantly fell within the 95% confidence limits of the null distributions under all comparisons, leading to non-rejection of the hypothesis of retained niche similarity being different than random (Figs 3b,c and S1b,c). Additionally, under the background of a geographic minimum convex polygon (MCP) with 50-km buffer zone, similar niche patterns were revealed (Fig. S2).

### Niche comparisons based on ENM predictions

For quantifying tolerance of environmental niche dimensions from the ENMs^41^, the largest overlap index of niche occupancy (*θ*) occurred in C-E for most variables considered separately (Fig. S3). By comparing the estimates of environmental suitability based on the outputs of ENMs, niche overlap metric of Schoener’s *D*, respectively, yielded values of 0.45 for the clade pair C-E; 0.17 for W-E; and 0.24 for W-C. According to the tests of niche equivalency via ENMtools^40^, the hypothesis that any clade pair is distributed in identical environmental space could be rejected (Mann-Whitney U tests, all *P* < 0.05; Fig. S4).

## Discussion

In this study, we use ENMs and multivariate niche analyses to elucidate environmental variations among clades of *Gynandropaa* frogs on the southeastern Qinghai-Tibetan Plateau (QTP). In particular, we address the question whether *Gynandropaa* clades conserved similar niches after divergence, which could provide insight on *Gynandropaa* frogs diversification. For this, we tested niche conservatism and the relation to lineage diversification based on up-to-date phylogeographic knowledge^25^,^27^,^38^. Our findings should therefore be interpreted in the light of our previous and ongoing studies of dicroglossid frogs (e.g. Hu *et al*.^29^, Huang *et al*.^38^), and allow exploring the information provided by ecological niche comparisons to understand amphibian diversification processes on the QTP.

**Figure 1:**
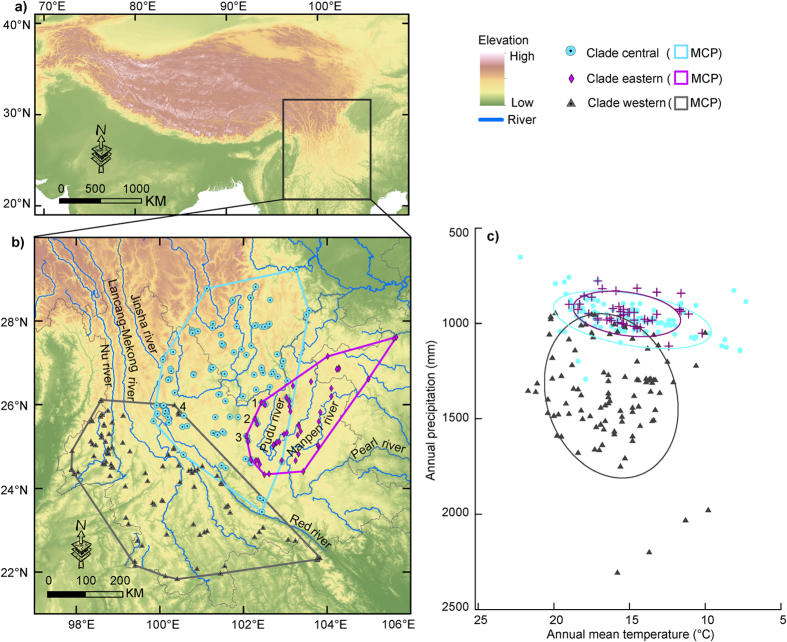
Distributions for the three clades of *Gynandropaa* frogs in geographical and environmental space. Panels (**a–c**) represent the geographical location of the study area on the Qinghai-Tibetan Plateau (**a**), occurrence records of the three clades in geographical space (**b**) and environmental space (**c**; annual mean temperature versus annual precipitation over this domain), respectively. In panel (**b**), a geographic minimum convex polygon (MCP) is defined for each clade; Arabic numerals: 1, Luquan; 2, Wuding; 3, Lufeng, and 4, Binchuan. In panel (**c**), ellipses are 75% of confidence sample. Panels (**a,b**) were drawn using ArcGIS 9.3 (ESRI, Redland, CA. URL http://www.esri.com/).

**Figure 2:**
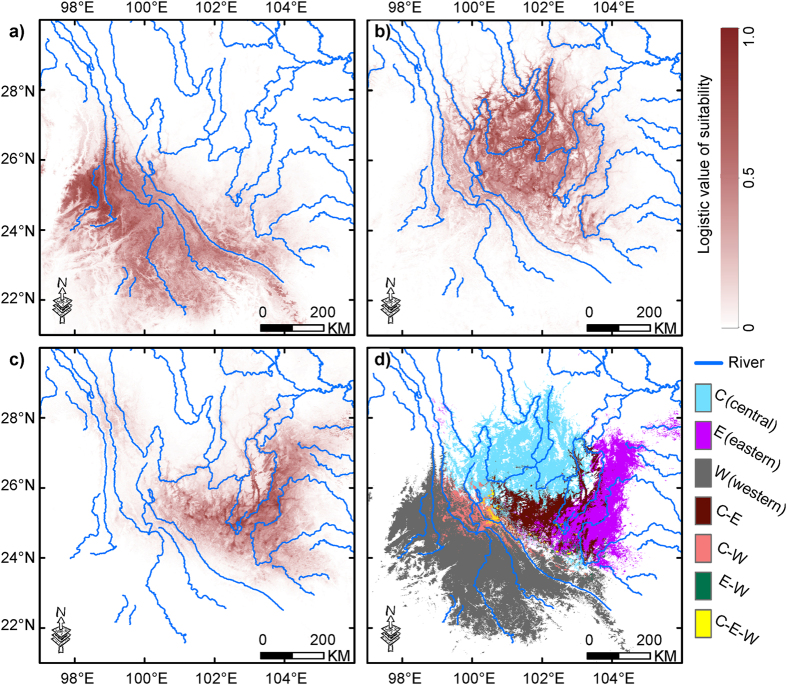
Predicted environmental suitability in geographical space for the three clades of *Gynandropaa* frogs. Panels (**a–c**) show predicted suitability (logistic probability) in geographical space for the clades western, central and eastern, respectively; (**d**), potential sympatric ranges, obtained by superimposing the presence/absence maps based on a logistic environmental suitability value representing the threshold indicating maximum training sensitivity plus specificity. All panels were drawn based on the projected distributions using ArcGIS 9.2 (ESRI, Redland, CA. URL http://www.esri.com/).

**Figure 3:**
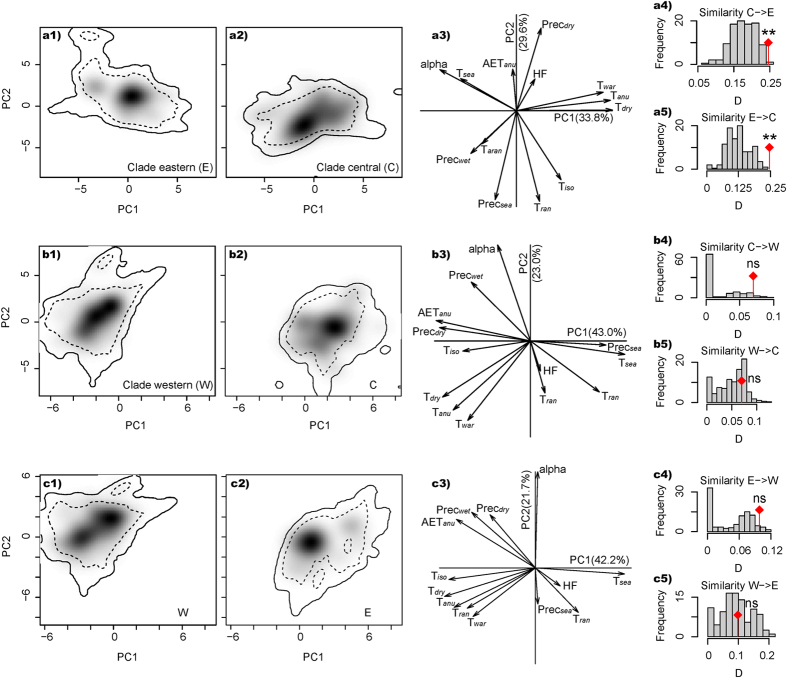
Niche of the clades of *Gynandropaa* frogs in relation to clade pairs in environmental space from a principal component analysis (PCA-env). Panels (**a–c**) represent the niche characteristics of the clade pairs C-E, C-W and W-E, respectively, along the two-first axes of the PCA. In panels (a1-2, b1-2, c1-2), grey shading shows the density of the occurrences of the clade by cell. The solid and dashed contour lines illustrate, respectively, 100% and 50% of the available (background) environment. The background area is delimited by each clade’s thresholded prediction from environmental niche modeling. Panels (a3, b3, c3) represent the contribution of the environmental variables on the first two axes of the PCA and the percentage of inertia explained by the two axes. Histograms (a4-5, b4-5, c4-5) show the observed niche overlap (D) between the two clades (bars with a diamond) and simulated niche overlaps (grey bars) on which tests of niche similarity are calculated. The significance of the tests is shown (ns, non-significant; **P < 0.05).

Although geographic regions of high environmental suitability for each clade are clearly delimited, some areas are predicted for multiple clades, allowing some potential areas of sympatry to be identified. Our models predict larger areas of sympatric occurrence of two clades in the clade pairs C-E and W-C than that in the pair W-E (Fig. 2d). These sympatric areas most likely satisfy partial environmental requirements for more than one clade simultaneously^42^. Accordingly, we could reject the hypothesis that any clade pair is distributed in strictly equivalent environmental space via tests of niche-equivalency^9^,^40^. Rather, our results show some evidence for niche conservatism as a whole, even though clade W goes relatively far beyond environmental tolerances of clades C and E with smaller observed niche overlap (Figs 1c, 3 and S1-3; Table S1). Despite this, our results suggest that widespread niche divergence among clades is lacking. These results are congruent with the expectation that closely related clades will not be equivalent in their environmental niches, but will typically be more similar than expected given the suites of environments available to them^43^. Simulated niche overlap values by PCA-env are generated by random shifts of the entire shape of the clade’s niche over the clade’s background area (the contour lines in Figs 3, S1 and S2) and provide a simpler environmental space (i.e. a linear combination of original predictors) in which niche differences are conserved^10^. Consequently, the results of PCA-env in E-space seem reliable for *Gynandropaa* frogs as the three clades occupy areas with dramatic variations in climate and topography on the southeastern QTP^24^.

Emergence of secondary contact can create niche differentiation when competition occurs between different taxa with similar ecological niches. This can result in the exclusion of taxa with weak competitive capability in some areas or environment^44^. Although previously isolated by the Paleo-Yangtze River, secondary contacts between clades C and E (in Luquan, Lufeng and Wuding; Fig. 1b) were caused by recent range expansions of these clades^25^. Clade C is considered to show evidence for introgressive capture of mitochondrial genomes via interspecific hybridization with clade E^25^,^27^. This suggests that competitive exclusion may play an important role. The clear evidence for niche conservatism in the pair C-E in the E-space is in accordance with their partially overlapping distributions^25^,^27^,^38^ and corresponding occupied climatic conditions (Fig. 1c). Due to low dispersal capability leading to significant genetic structure and restricted gene flow among clades after geographic separation^25^, Wang^27^ assumed that genetic isolation between them has arisen and was maintained even in the subsequent secondary contacts^27^. Moreover, for clade pairs C-W and W-E, failure to reject the null hypothesis of PCA-env can provide a hint of no significant differentiation^9^; the difference in the *P*-values between reciprocal directions for these clade pairs suggest that one niche is likely partially nested within the other (Figs 3b,c and S2b,c). Still, the niches for these clade pairs are clearly not strictly equivalent (Fig. S4) and indicate that clades of *Gynandropaa* frogs are likely to have subsequently adapted to different environments.

Both geographic and ecological dimensions may contribute to diversification processes^45^. For *Gynandropaa* frogs, Wang^27^ revealed the first geographic barrier as the Red river, always between clades W and E or C, while the Paleo-Yangtze River that initially isolated clades C and E lost its barrier-effect when it reversed to flow eastward^46^. The phylogeography of this group demonstrates that the long-term genetic isolation among clades is corroborated by the results of analysis of molecular variance with significant genetic structure and isolated in different montane streams, despite opportunities for geographic contact and hybridization with each other in the past^25^,^27^. Environmental conditions during the Middle Pleistocene accompanying dramatic climatic oscillations permitted extensively historical range expansions and the mixing of the gene pools in the secondary contact of previously allopatric populations from different clades^25^,^27^. Thus, they seem to have maintained separate evolutionary trajectories in the face of historical opportunities for secondary contact. Additionally, the estimated diversification times among clades based on the molecular clock^25^ approximately corresponded to the most significant geological changes in the Yunnan Plateau occurred from the Pliocene to the Early Pleistocene^47^, when a third uplifting on QTP occurred more strongly and frequently^46^. These geological changes must have led to repeated isolation and fragmentation events that could have driven the vicariance of *Gynandropaa* frogs, leading overall to niche conservation among clades. Based on these findings, and although the same patterns of niche conservation could be obtained through sympatric speciation followed by dispersal and range expansion, it is not the most parsimonious explanation given our current knowledge and seems thus rather unlikely to have occurred in *Gynandropaa* frogs. Support is provided, in particular, by the facts that, within sympatric areas, individuals from different clades at the same localities can be classified by mitochondrial DNA, and given the deep divergence in mtDNA between clades whose gene flow is strongly restricted with suggested introgression^25^,^27^. It seems likely that periods of allopatric isolation have been accompanied by the loss of interbreeding capability among clades. Our niche conservatism findings may thus be seen as a ‘symptom’ supporting the hypothesis of allopatric speciation processes^25^,^27^, with clades largely isolated on opposite sides of barriers, but similar ecological conditions prevailing on all sides (Fig. 1). Accordingly, this study is generally compatible with the conclusion that niche divergence is likely not the major driver of clade diversification process in *Gynandropaa*, while the three clades have seemingly originated through vicariance events^27^ associated with evolutionary conservatism in their environmental tolerances. It should be noted, however, that the niches fitted here were rather measured at the macro-environmental scale, and one cannot exclude from these results that niche differentiations may have occurred between clades at a more micro-environmental scale^48^,^49^,^50^. In fact, both niche conservatism and niche divergence could be observed following a geographic separation depending on the geographic scale considered, which will also depend on the environment on all sides of the barriers. If the environments are rather similar, the niches are likely to remain the same for the two clades, whereas if the environments differ, the populations on each side of the barrier are likely to see their niche shift toward a different centroid, and thus diverge.

This study illustrates a framework for studying the role of ecological niche in diversification processes and over a broad range of taxa. Results from our similarity and equivalency niche tests support that niche conservatism may be seen as a signature of between-clade allopatric speciation in *Gynandropaa* frogs but do not exclude slight niche divergences or more pronounced niche divergence at finer, unmeasured scales. Even though we focused on a small radiation of spiny frogs, the methods employed here for amphibians may be generalized to and help explain patterns of diversity for other vertebrates or even plants in areas on and adjacent to the QTP. Since previous field surveys, molecular analyses and conservation priorities have been heavily biased toward mammals and birds^26^,^28^,^51^,^52^, we infer that genetic diversity and endemism of amphibians on the QTP have been substantially underestimated. In a world of ever-accelerating environmental changes^53^,^54^,^55^,^56^, this study may also be useful in guiding research with regard to lineage diversification, as well as conservation in other threatened global hotspots or similarly complex plateau ecosystems.

## Methods

### Digital occurrence records

Occurrence data were gathered from direct observations during extensive field expeditions (2003–2011). Further data was obtained in the form of localities with geo-coordinates from the literature^25^,^27^ and from georeferenced specimen records in the Herpetological Museum of the Chengdu Institute of Biology, CAS. For clade identifications, due to the phenotypic similarity, we assigned individuals from the potential sympatry to specific clade based on the phylogenetic analyses using mitochondrial DNA sequences^25^,^27^,^38^; additionally, we identified individuals from allopatric areas to clade using morphological characters and geographic information^38^. Occurrences were then assigned to the three clades (i.e. W, C and E)^25^,^27^,^38^, covering the whole distribution range of *Gynandropaa* frogs (Fig. 1b; see also the literature^25^,^26^,^27^,^38^). We treated all occurrences equally without consideration of the population size, and double-checked occurrences using spreadsheets and GIS to detect duplicates and possible georeferencing errors. We then filtered occurrences spatially to ensure that only one record was left per grid cell for each clade at a spatial resolution of 30 arc-seconds with trimming duplicate occurrences in ENMtools^40^. Our final dataset comprised 100 georeferenced occurrences for W, 115 for C and 47 for E (Fig. 1b).

### Environmental variables

To characterize environmental heterogeneity across the distribution range for *Gynandropaa*, we initially compiled 34 environmental variables (Appendix S1; Table S2). These included 19 bioclimatic variables^57^ and other 15 macro-environmental variables referring to climate, hydrology, soil, topography, land cover, and human impact recognized as important factors potentially shaping distribution limits of wildlife. As strong colinearity between environmental variables could inflate model accuracy in ENM, it is important to minimize correlations among variables using dimension-reduction techniques (e.g. correlation analysis and/or clustering algorithms)^58^,^59^. Hence, integrating the results of Pearson’s correlation tests (certain temperature/precipitation variables being removed owing to high correlations with other temperature/precipitation variables with the threshold of correlation coefficients of |*r*| > 0.8^60^) and a jackknife analysis (retaining variables with the higher value when used in isolation^39^), we reduced the number of predictor variables. When using the jackknife procedure to evaluate the relative importance of each variable, the model was re-run by excluding each variable in turn; then a model was created using each variable in isolation. We retained 14 variables (Appendix S1; Fig. S5) that included T_*anu*_, T_*ran*_ (mean monthly temperature range), T_*iso*_, T_*sea*_ (temperature seasonality), T_*aran*_ (temperature annual range), T_*dry*_, T_*war*_, Prec_*wet*_, Prec_*dry*_, Prec_*sea*_ (precipitation seasonality) from the Worldclim database^57^, AET_*anu*_, alpha from the Consortium for Spatial Information (http://www.cgiar-csi.org), land-cover from the Global Land Cover 2000 database (http://gem.jrc.ec.europa.eu/products/glc2000/glc2000.php), and HF (human footprint index)^61^. All variables were at a spatial resolution of 30 arc-seconds (~1 km). These variables reflected meaningful environmental conditions to which frogs are exposed and which are known to impose constraints on the physiology and survival of amphibian species^62^,^63^; climatic factors (e.g. temperature extremes and the seasonality of precipitation) are often used to model the geographical distributions of amphibians^54^,^55^.

### Fitting environmental niche modeling

We developed ENMs for the three *Gynandropaa* clades (W, C and E) using Maxent 3.3.3k^39^. The Maxent model works by optimizing a set of constraints representing the incomplete information on distribution and evaluating the environmental suitability of each grid cell within the study area^39^,^64^. The study area here was defined according to the distribution range of *Gynandropaa* frogs (97–106°E and 21–30°N; Fig. 1a,b)^25^,^38^. Maxent has been shown to have good predictive performance across various applications^65^. We followed the default settings for Maxent models^66^. The default settings have been justified provided that they have been validated over a wide range of species using different sets of environmental variables in various regions of the world, and were shown to achieve good performance^66^. These settings were appropriate for our modeling efforts with the same environmental variables in the same study region (see also^67^,^68^). We generated 100 replicates using the bootstrap method with 75% of occurrences used for model training and 25% for testing. The importance of each variable was evaluated by the jackknife analyses. We used the AUC metric that is a threshold-independent measure as an index of discrimination capacity^69^, and calculated the average value of replicates. Use of AUC analyses with presence-only evaluation datasets has been clarified and justified for the classification of presence versus random, using information regarding the background of the study region rather than pseudo-absences and avoiding commission error^39^,^58^,^70^. Logistic output format was selected for revealing the predicted environmental suitability due to being easily interpretable, with values ranging from 0 (lowest) to 1 (highest)^66^. Additionally, when analyzing niche overlap referring to Evans *et al*.^41^, we re-ran the Maxent model to obtain predicted suitability of “raw probabilities”. Choice of a suitability threshold can have a great effect on predicted maps, and there is still no consensus on the selection of optimal threshold^58^,^71^. Continuous suitability outputs were thus thresholded using the threshold indicating maximum training sensitivity plus specificity which is considered as a more robust approach^71^.

### Comparing niche overlap based on observed occurrences in E-space

Both actual niche differences and spatially-autocorrelated environmental variations can result in niche differentiation between taxa^20^. To estimate niche differentiations, the niche similarity tests can rely on either ordination techniques or ENMs^9^,^10^,^23^. The niche similarity test differs from the niche equivalency test since the former assesses whether observed niche overlaps between clades are different than simulated overlaps between niches of the same shapes and sizes but randomly centered in the background^9^,^10^. This test distinguishes the differentiations resulting from simple spatial autocorrelation caused by geographic distance from true niche differences that occurs because two species occupy different habitats^19^,^20^,^72^. However, the niche similarity test using geographical projections of niches in G-space could prove problematic due to measured niche overlap likely varying with the changing of the extent and distribution of environmental gradients in the study area^9^,^10^. Accordingly, we used the niche similarity test based on the ordination technique in E-space^10^. Based on this ordination technique, several shortcomings, i.e. accounting for biases introduced by spatial resolution (grid size), making optimal use of both geographical and environmental spaces, and correcting observed occurrence densities for each region in light of the availability of environmental space, can be overcome when quantifying niche differences^10^.

We employed the approach of PCA-env which can most accurately retrieve the simulated level of niche overlap among ordination techniques considered and without substantial bias^10^. PCA-env calculates the occurrence density and environmental factor density along environmental (principal component) axes for each cell using a kernel smoothing method and then uses the density of both occurrences and environmental variables to measure niche overlap along these axes. Species occurrences are then projected onto the gridded environmental space (at a resolution of 100 × 100 cells) of the first three axes for ordinations such as principal components analysis (PCA) calculated with the selected environmental variables (excluding the categorical variable of land-cover). An unbiased estimate of the Schoener’s *D* metric was calculated for our data using smoothed densities from a kernel density function to measure niche overlap between clades that is ensured to be independent of the resolution of the grid (see Fig. S1b in Broennimann *et al*.^10^). Statistical confidence in niche overlap values was then tested through a one-sided niche-similarity test^10^. The observed overlap values greater than the simulated values indicate that niches of the clade pair under comparison are more similar than random. The background area used in the similarity test should reflect the area accessible to the organism^9^,^72^. To test whether our results are robust to different selections of the background, referring to Theodoridis *et al*.^73^, we used two different approaches. We first delimited the background using each clade’s thresholded prediction from ENMs^71^. Next, we used the background defined by a MCP with 50-km buffer zone that circumscribed occurrences of each clade^40^,^73^, in ArcGIS 9.3 (ESRI, Redlands, CA). Under the background from the ENMs binary predictions, the first three axes explained 83.8%, 81.1% and 78.6% of the overall variance for the pairs C-E, C-W and W-E, respectively. Under the background from MCPs, the first three axes explained 79.7%, 79.5% and 76.7% of the overall variance for the pairs C-E, C-W and W-E, respectively. All statistical analyses were performed in R 3.0.2^74^ using scripts in Broennimann *et al*.^10^, now available in the ‘ecospat’ R package^75^.

### Niche comparisons based on ENM predictions

We used two complementary approaches to explore niche comparison among the three clades (W, C and E) based on ENM predictions. Firstly, to quantify the niche breadth in environmental dimensions for each clade, we integrated environmental suitability from Maxent with respect to each original variable to produce unit area histograms of suitability that illustrate the predicted occupancy of each variable. We quantified the overlap index of niche occupancy (*θ*) in each variable (excluding the categorical variable of land-cover) by comparing predicted environmental occupancy profiles following Evans *et al*.^41^, with the formula 
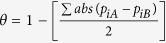
, where *p*_*i*A_ and *p*_*i*B_ are total predicted suitability at a given value (*i*) of a particular variable for clades A and B, respectively^41^.

Then, we tested the null hypothesis of niche equivalency^9^,^40^. This test is based on the metric of niche overlap (i.e. Schoener’s *D*), ranging from 0 (no overlap) to 1 (complete overlap), that compare the estimates of environmental suitability for each grid cell in the study area after normalizing each clade’s ENM^9^,^40^. It begins by pooling all occurrences for a pair of clades, randomly splitting into two datasets with the same number of occurrences as in the two original datasets^40^. For each pseudoreplicate of this process, ENMTools uses the predictions of environmental suitability for each clade to calculate similarity metrics. Then, a distribution of overlap scores between clades drawn from a shared distribution is obtained, assuming that the clades are interchangeable in their use of niche space. This process is repeated 100 times (to ensure that the null hypothesis can be rejected with high confidence) and a pseudoreplicated null distribution of simulated values is constructed using ENMTools v1.3^40^. The null hypothesis of niche equivalency is rejected when the observed values for similarity metrics are significantly different from the pseudoreplicated data sets^40^.

## Additional Information

**How to cite this article**: Hu, J. *et al*. Niche conservatism in *Gynandropaa* frogs on the southeastern Qinghai-Tibetan Plateau. *Sci. Rep.* **6**, 32624; doi: 10.1038/srep32624 (2016).

## Supplementary Material

Supplementary Information
