# Supplementary material for: Niche conservatism in Gynandropaa frogs on the southeastern Qinghai-Tibetan Plateau
Source: Sci Rep. 2016 Sep 7;6:32624. doi: 10.1038/srep32624 (PMC5013482; doi:10.1038/srep32624)
Supplement: Supplementary Information [file srep32624-s1.pdf]

Niche conservatism in *Gynandropaa* frogs on the southeastern Qinghai-Tibetan Plateau

Junhua Hu[1*], Olivier Broennimann[2], Antoine Guisan[2], Bin Wang[1], Yan Huang[1] & Jianping Jiang[1*]

[1]*Chengdu Institute of Biology, Chinese Academy of Sciences, Chengdu* 610041*, China*

[2]*Department of Ecology and Evolution, University of Lausanne, 1015 Lausanne, Switzerland*

Supporting Information

Appendix S1

Preparing for environmental variables

To characterize the environmental heterogeneity across the distribution range of *Gynandropaa*, we initially compiled 34 environmental variables (Table S2). These included 19 bioclimatic variables derived from the Worldclim database[1] and other 15 macro-environmental variables recognized as important factors potentially shaping distribution limits of wildlife. The bioclimatic variables describe estimates of annual means, degrees of seasonality, and potentially biologically limiting extremes in temperature and precipitation.

To consider representative soil-water balance and soil property, we obtained other environmental variables from the Center for Sustainability and the Global Environment (http://www.sage.wisc.edu/atlas/index.php), the Consortium for Spatial Information (http://www.cgiar-csi.org) (Table S2). We obtained land cover data from the Global Land Cover 2000 database[2]. Additionally, to incorporate the potential anthropological impacts, we used the human footprint index (HF)[3]. We also obtained variables representing topography from the USGS's Hydro1K dataset (Table S2). The variables from the Center for Sustainability and the Global Environment (at a spatial resolution of 0.5 arc-degree) were resampled to a resolution of 30 arc-seconds using a bilinear interpolation function which is considered to be more realistic than the simpler nearest-neighbor method[4]. All other variables were at a spatial resolution of 30 arc-seconds.

It is important to minimize correlation among variables and to remove highly correlated variables using dimension reduction methods (e.g. correlation analysis and clustering algorithms)[5, 6]. We reduced the variables by integrating the results of Pearson's correlation tests and jackknife analysis. Consequently, 14 relatively independent and important variables were filtered owing to low correlations with other variable pairs ($|r| < 0.8$) and the higher value in the regularized gain to

the Maxent model (Fig. S5)[4]. The selected variables included annual mean temperature (T_{anu}), mean monthly temperature range (T_{ran}), isothermality (T_{iso}), temperature seasonality (T_{sea}), temperature annual range (T_{aran}), mean temperature of the driest quarter (T_{dry}), mean temperature of the warmest quarter (T_{war}), precipitation of the wettest month (Prec_{wet}), precipitation of the driest month (Prec_{dry}), precipitation seasonality (Prec_{sea}), AET_{anu}, alpha, land-cover, and HF (Table S2).

References

1. Hijmans, R. J., Cameron, S. E., Parra, J. L., Jones, P. G., Jarvis, A. Very high resolution interpolated climate surfaces for global land areas. *International Journal of Climatology* **25**, 1965-1978 (2005).
2. GLC. Global Land Cover 2000 database. (ed^(eds). European Commission, Joint Research Centre (2003).
3. Sanderson, E. W., Jaiteh, M., Levy, M. A., Redford, K. H., Wannebo, A. V., Woolmer, G. The human footprint and the last of the wild. *Bioscience* **52**, 891-904 (2002).
4. Phillips, S. J., Anderson, R. P., Schapire, R. E. Maximum entropy modeling of species geographic distributions. *Ecol. Modell.* **190**, 231-259 (2006).
5. Hu, J., Jiang, Z. Predicting the potential distribution of the endangered Przewalski's gazelle. *Journal of Zoology* **282**, 54-63 (2010).
6. Merow, C., Smith, M. J., Silander, J. A. A practical guide to MaxEnt for modeling species' distributions: what it does, and why inputs and settings matter. *Ecography* **36**, 1058-1069 (2013).

Table S1 Weighted mean values for the environmental variables given the predicted niche models for the three clades in *Gynandropaa* frogs (T = temperature in °C; P = precipitation in mm)

Clades	T_{anu}	T_{ran}	T_{iso}	T_{sea}	T_{aran}	T_{dry}	T_{war}	$Prec_{wet}$	$Prec_{dry}$	$Prec_{sea}$	HF	AET_{anu}	Alpha
western	16.9	11.4	49.9	3955.8	22.9	12.2	21.0	288.1	14.4	83.5	28.2	934.2	72.4
central	15.4	11.7	47.4	4581.0	24.6	9.4	20.4	217.0	8.1	94.4	32.4	785.4	63.5
eastern	15.7	10.7	44.9	4653.0	23.6	9.8	20.7	196.9	11.7	85.6	35.0	793.1	66.2

*T_{anu} (annual mean temperature), T_{ran} (mean monthly temperature range), T_{iso} (isothermality), T_{sea} (temperature seasonality), T_{aran} (temperature annual range), T_{dry} (mean temperature of the driest quarter), T_{war} (mean temperature of the warmest quarter), $Prec_{wet}$ (precipitation of the wettest month), $Prec_{dry}$ (precipitation of the driest month), $Prec_{sea}$ (precipitation seasonality), HF (human footprint index), AET_{anu} (annual actual evapotranspiration), alpha (Priestley-Talor alpha coefficient).

Table S2 Environmental variables compiled to fit environmental niche modeling and test niche conservatism for *Gynandropaa* frogs.

Abbreviation	Variable name	Included in models (Yes/No)	Explanations	Citation
bio01	Annual mean temperature	Yes	-	Worldclim database
bio02	Mean diurnal range	Yes	Mean of monthly (max temp - min temp)	Worldclim database
bio03	Isothermality	Yes	Isothermality (bio02/ bio07) (*100)	Worldclim database
bio04	Temperature seasonality	Yes	Temperature seasonality (standard deviation*100)	Worldclim database
bio05	Max temperature of the warmest month	No	-	Worldclim database
bio06	Min temperature of the coldest month	No	-	Worldclim database
bio07	Temperature annual range	Yes	Temperature annual range (bio05-bio06)	Worldclim database
bio08	Mean temperature of the wettest quarter	No	-	Worldclim database
bio09	Mean temperature of the driest quarter	Yes	-	Worldclim database
bio10	Mean temperature of the warmest quarter	Yes	-	Worldclim database
bio11	Mean temperature of the coldest quarter	No	-	Worldclim database
bio12	Annual precipitation	No	-	Worldclim database
bio13	Precipitation of the wettest month	Yes	-	Worldclim database
bio14	Precipitation of the driest month	Yes	-	Worldclim database
bio15	Precipitation seasonality	Yes	Precipitation seasonality (coefficient of variation)	Worldclim database
bio16	Precipitation of the wettest quarter	No	-	Worldclim database
bio17	Precipitation of the driest quarter	No	-	Worldclim database
bio18	Precipitation of the warmest quarter	No	-	Worldclim database
bio19	Precipitation of the coldest quarter	No	-	Worldclim database
GDD	Growing degree days	No	Total number of growing degree days (based on a 5°C base temperature) in each grid cell over one year	Atlas of the Biosphere
NPP	Net primary productivity	No	Total net primary productivity in each grid cell over one year	Atlas of the Biosphere
SM	Soil moisture	No	Average amount of water in the soil of a grid cell	Atlas of the Biosphere
SC	Soil organic carbon	No	Total mass of soil carbon (to one meter) contained in a grid cell	Atlas of the Biosphere
SpH	Soil pH	No	The pH of the soil in a grid cell	Atlas of the Biosphere
AET_{anu}	Annual actual evapotranspiration	Yes	The effective quantity of water that is removed from the soil due to evaporation and transpiration processes	Consortium for Spatial Information
AI	Annual aridity index	No	The ratio of mean annual precipitation over annual potential evapotranspiration	Consortium for Spatial Information
SolRad	Extraterrestrial solar radiation	No	Radiation on top of atmosphere	Consortium for Spatial Information
alpha	Priestley-Talor alpha coefficient	Yes	The ratio of annual actual evapotranspiration over annual potential evapotranspiration	Consortium for Spatial Information
PET	Annual potential evapotranspiration	No	A measure of the ability of the atmosphere to remove water through evapotranspiration processes	Consortium for Spatial Information
landcov	Land-cover	Yes	Representation of vegetation land cover into a limited number of classes	GLC 2003
HF	Human footprint index	Yes	An estimate of human influence based on human settlements, land transformation, accessibility and infrastructure data	Sanderson et al. 2002
aspect	Aspect	No	The direction of maximum rate of change in the elevations between each cell and its eight neighbors.	Hydro1K dataset
slope	Slope	No	The maximum change in the elevations between each cell and its eight neighbors	Hydro1K dataset
CTI	Compound Topographic Index	No	A function of the upstream contributing area and the slope of the landscape	Hydro1K dataset

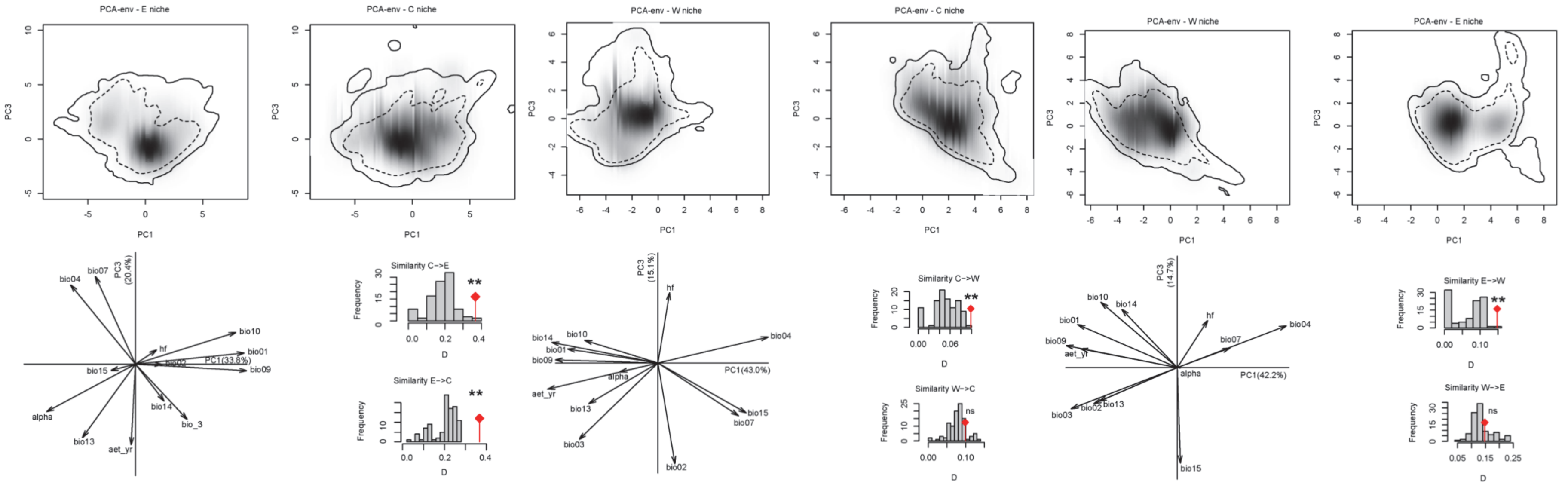
PCA-env - E niche
PCA-env - C niche
PCA-env - W niche
PCA-env - C niche
PCA-env - W niche
PCA-env - E niche
Similarity C->E
Similarity E->C
Similarity C->W
Similarity W->C
Similarity E->W
Similarity W->E
PC1(33.8%)
PC3 (20.4%)
PC1(43.0%)
PC3 (15.1%)
PC1(42.2%)
PC3 (14.7%)

67

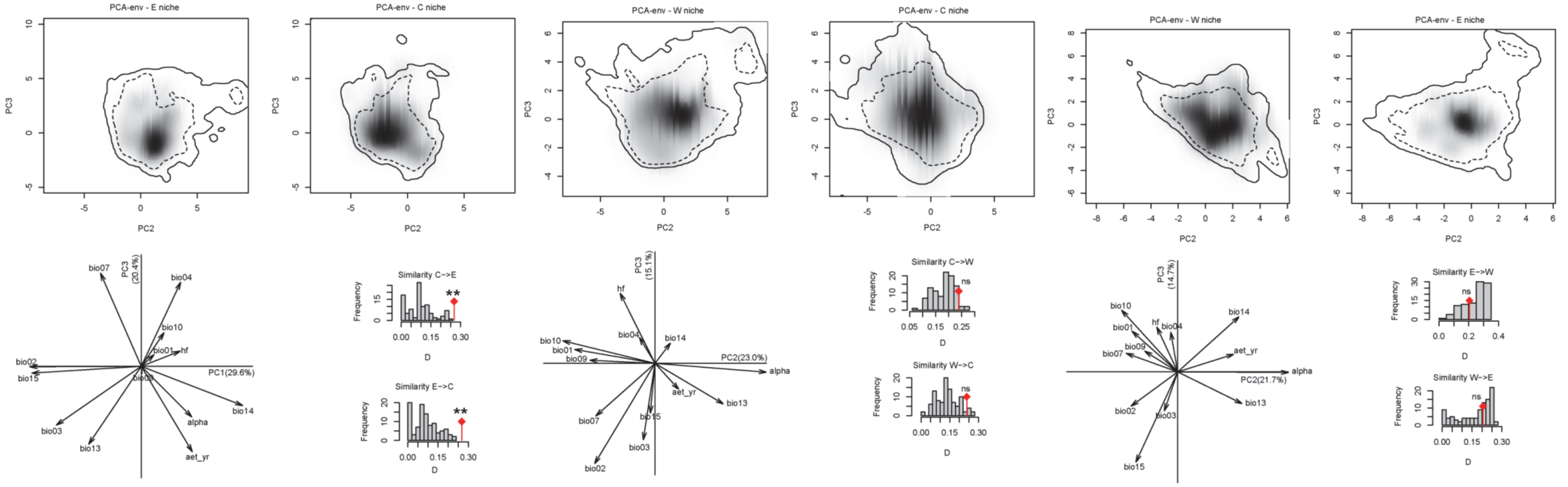
PCA-env - E niche
PCA-env - C niche
PCA-env - W niche
PCA-env - C niche
PCA-env - W niche
PCA-env - E niche
Similarity C->E
Similarity E->C
Similarity C->W
Similarity W->C
Similarity E->W
Similarity W->E
PC1(29.6%)
PC3 (20.4%)
PC2(23.0%)
PC3 (15.1%)
PC2(21.7%)
PC3 (14.7%)

68

Figure S1 Niche of the clades of *Gynandropaa* frogs in relation to clade pairs in environmental space from a principal component analysis (PCA-env) under the background of the ENMs binary predictions. Panels show the niche characteristics of the clade pairs C-E, C-W and W-E, respectively, along the two corresponding axes of the PCA (PC1 *vs*. PC3 & PC2 *vs*. PC3). Grey shading shows the density of the occurrences of the clade by cell; the solid and dashed contour lines illustrate, respectively, 100% and 50% of the available (background) environment. The contribution of the environmental variables on the two corresponding axes of the PCA and the percentage of inertia explained by the two axes are also represented. Histograms show the observed niche overlap (D) between the two clades (bars with a diamond) and simulated niche overlaps (grey bars) on which tests of niche similarity are calculated. The significance of the tests is shown (ns, non-significant; ** $P < 0.05$).

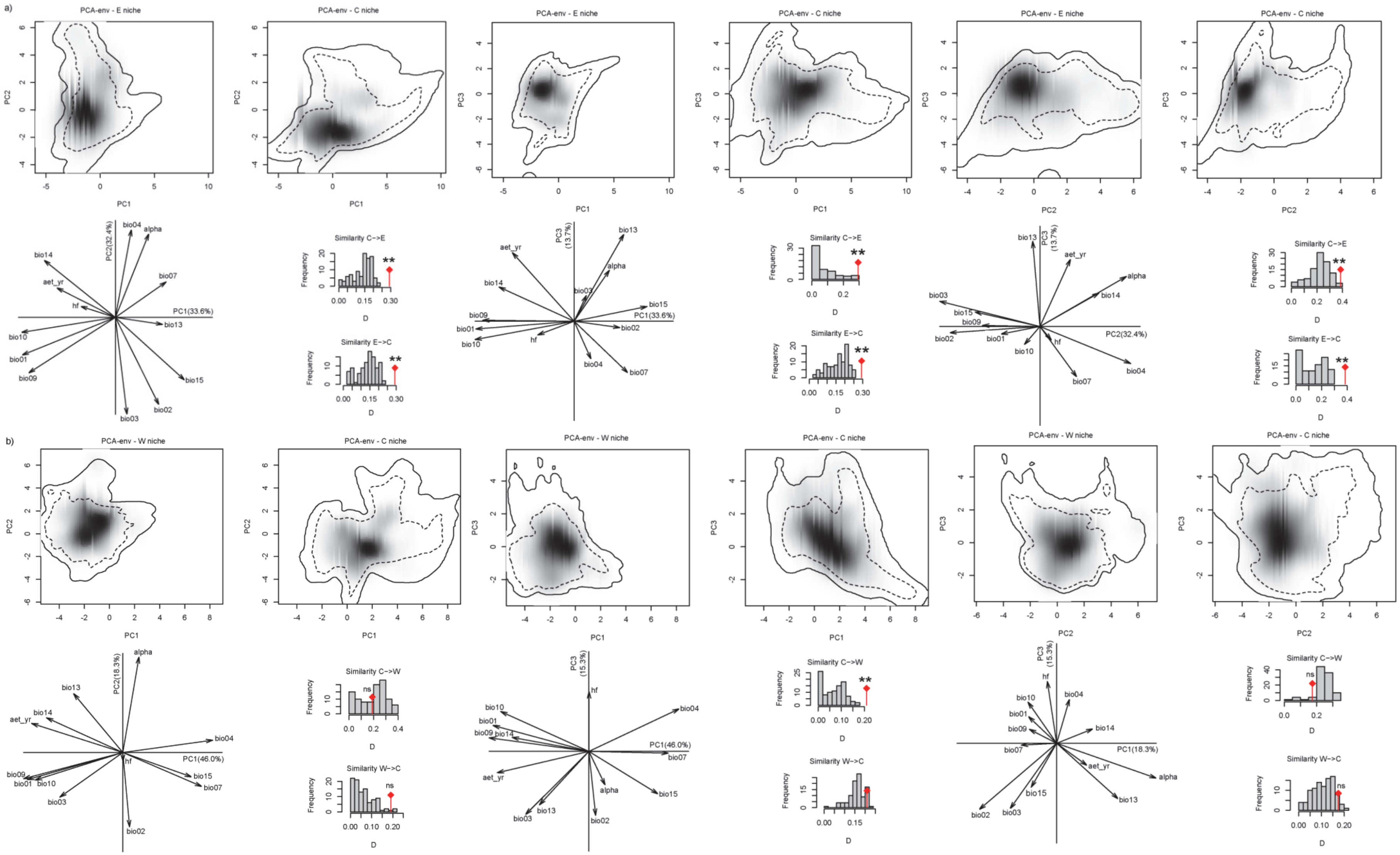
a)
PCA-env - E niche
PCA-env - C niche
PCA-env - E niche
PCA-env - C niche
PCA-env - E niche
PCA-env - C niche
PC1
PC2
PC3
PC1(33.6%)
PC2(32.4%)
PC3 (13.7%)
Similarity C->E
Similarity E->C
Frequency
D
**
b)
PCA-env - W niche
PCA-env - C niche
PCA-env - W niche
PCA-env - C niche
PCA-env - W niche
PCA-env - C niche
PC1(46.0%)
PC2(18.3%)
PC3 (15.3%)
PC1(18.3%)
Similarity C->W
Similarity W->C
ns
bio01
bio02
bio03
bio04
bio07
bio09
bio10
bio13
bio14
bio15
alpha
aet_yr
hf

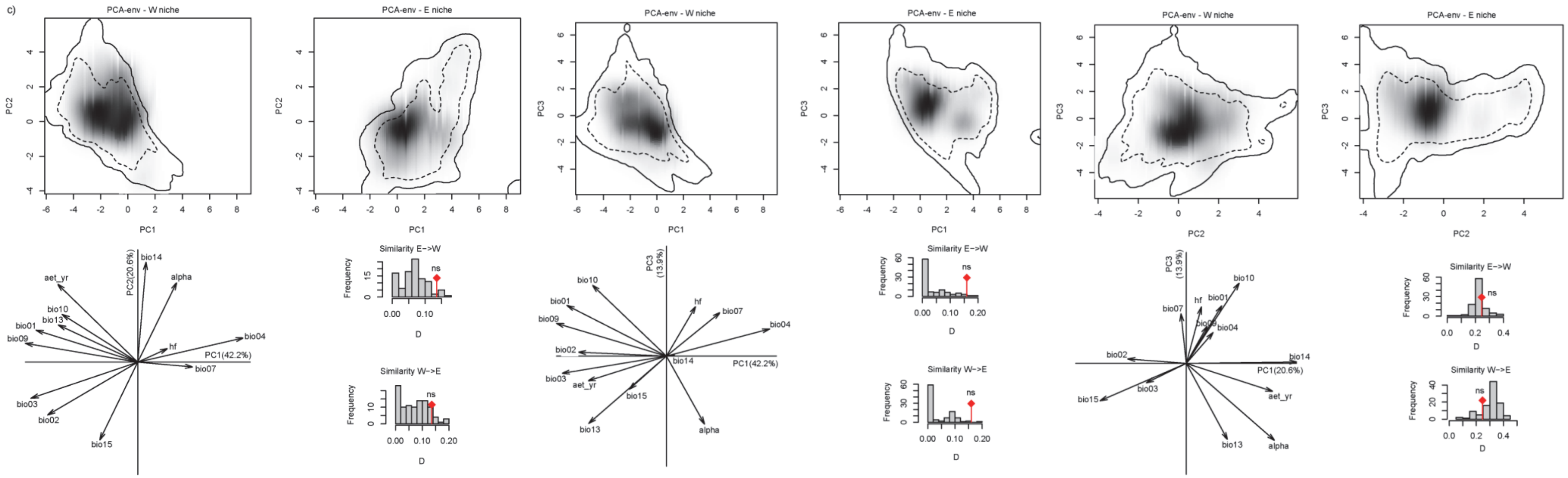

Figure S2 Niche of the clades of *Gynandropaa* frogs in relation to clade pairs in environmental space from a principal component analysis (PCA-env) under the background of a geographic minimum convex polygon with 50-km buffer zone around occurrences. Panels (a-c) represent the niche characteristics of the clade pairs C-E, C-W and W-E, respectively, along the three-first axes of the PCA. Grey shading shows the density of the occurrences of the clade by cell; the solid and dashed contour lines illustrate, respectively, 100% and 50% of the available (background) environment. The contribution of the environmental variables on the two corresponding axes of the PCA and the percentage of inertia explained by the two axes are also represented. Histograms show the observed niche overlap (D) between the two clades (bars with a diamond) and simulated niche overlaps (grey bars) on which tests of niche similarity are calculated. The significance of the tests is shown (ns, non-significant; ** $P < 0.05$).

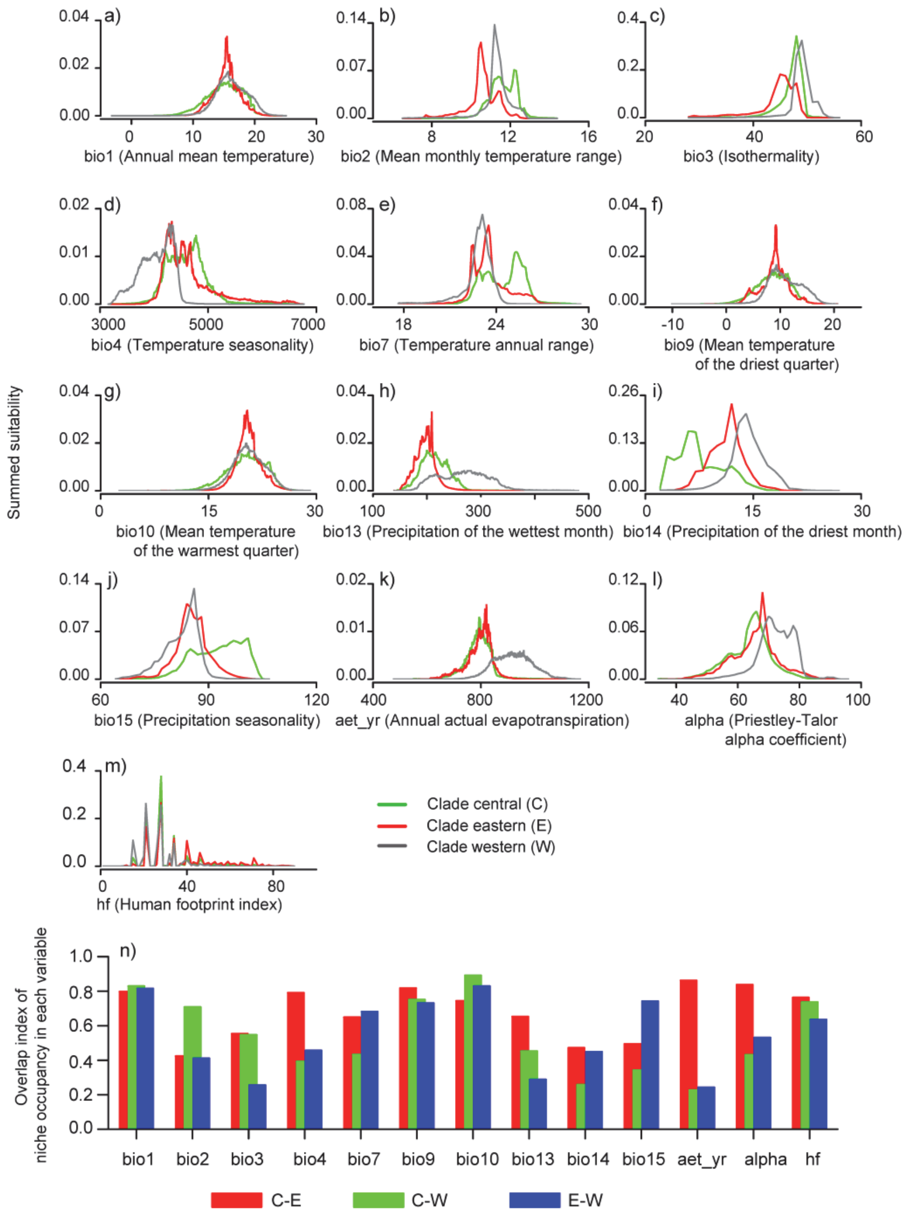

Figure S3 Predicted niche occupancy with respect to each environmental variable (panels a-m) for the three clades of *Gynandropaa* frogs and the overlap index of niche occupancy in each variable between clades (panel n). Predicted suitability is summed according to the environmental data with which it is associated. Suitability is rescaled to detect the difference among clades for each variable.

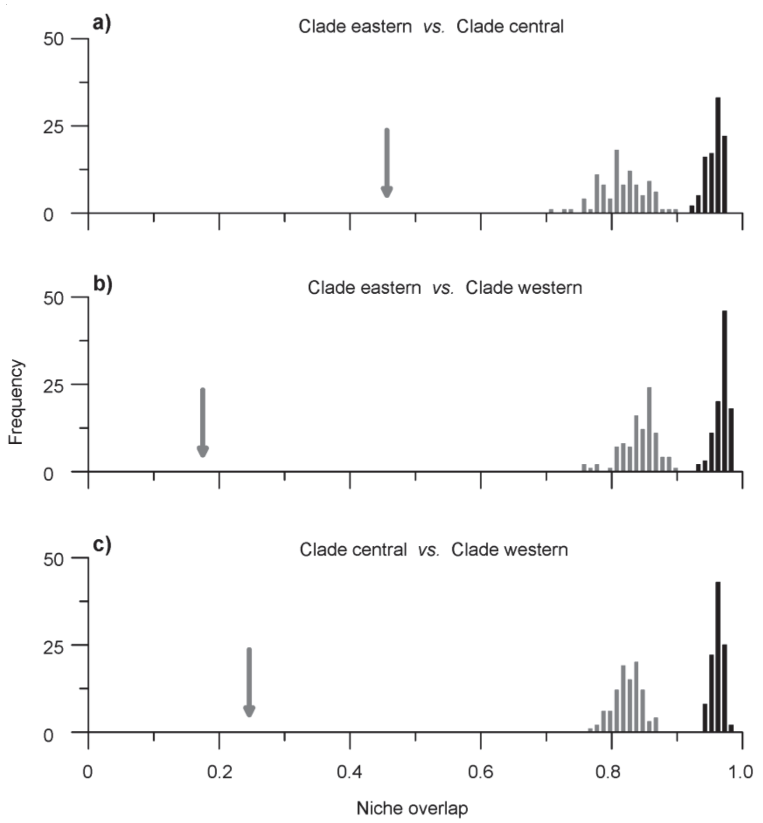

Figure S4 Results of the niche identity test for the three clades of *Gynandropaa* frogs. The vertical arrows in each panel represent the actual niche-overlap values for Schoener's *D* and that are compared to the null distributions for the corresponding clade pair. The histograms represent the distribution of niche similarities obtained from pairs of pseudo-niches constructed with the randomization of occurrence records of the two clades.

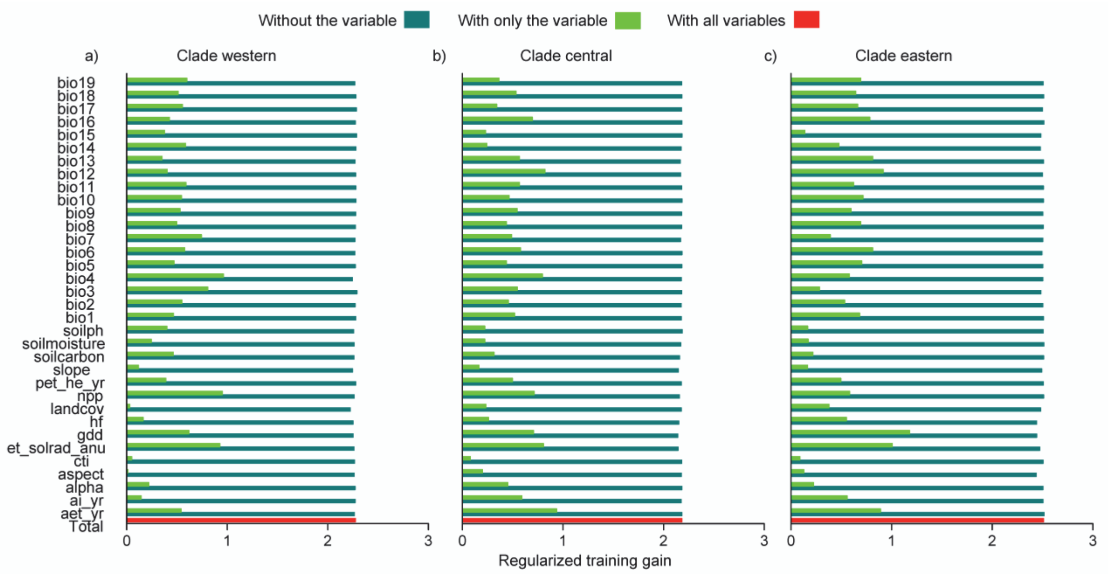

Figure S5 The jackknife test of variable importance for the three clades of *Gynandropaa* frogs.
